# Role of Centromeric and Telomeric Haplotypes of Killer-Cell Immunoglobulin-Like Receptors (KIRs) in Disease Susceptibility: A Research Review

**DOI:** 10.7759/cureus.83728

**Published:** 2025-05-08

**Authors:** Ehab A Elagab, Abdelrahman M Ibrahim, Amal Shediwah, Saad M Alqahtani, Sumaia M TalbAllah

**Affiliations:** 1 Department of Pathology, College of Medicine, Najran University, Najran, SAU

**Keywords:** centromeric kirs, disease susceptibility, kirs, nk cells, telomeric kirs

## Abstract

The immune regulatory functions of killer-cell immunoglobulin-like receptors (KIRs) become important through their ability to control natural killer (NK) cell function by interacting with human leukocyte antigen (HLA) molecules. Increasing scientific evidence indicates KIR haplotypes exist in two sets, namely, centromeric (Cen) and telomeric (Tel), which deliver unique effects to immune response function. Unlike traditional A/B KIR haplotype classification, this review emphasizes Cen and Tel subdivisions to provide a more nuanced understanding of gene linkage, functional diversity, and their relevance to autoimmune diseases, infections, transplantation outcomes, and pregnancy complications. It focuses on the coexpression of specific KIR genes driven by linkage disequilibrium, deliberately excluding studies limited to individual KIR alleles or classical haplotypes. Certain KIR configurations demonstrate significant influence on diseases through disease risk and also impact immune tolerance processes as well as resulting clinical outcomes. Our investigation through the literature revealed the relationship between Cen and Tel KIR haplotypes and rheumatoid arthritis, systemic lupus erythematosus (SLE), syphilis, and both hematopoietic stem cell transplantation (HSCT) and recurrent pregnancy loss (RPL). Researchers documented how Cen and Tel KIRs operate as either activating or inhibitory receptors in their study. However, few researches have studied the effect of Cen-Tel KIR haplotypes on the occurrence of diseases. There is a pressing need for research to focus on comprehensive KIR haplotyping analysis, as it holds the potential to enhance transplantation risk prediction, advance understanding in reproductive biology, uncover the pathogenesis of certain infectious diseases, and shed light on autoimmune-related disorders. Ultimately, the insights gained from Cen-Tel KIR haplotype analysis may serve as a foundation for developing targeted immunotherapeutics and personalized treatment strategies.

## Introduction and background

The polymorphic killer-cell immunoglobulin-like receptors (KIRs) function as surface receptors of natural killer (NK) cells and specific T cells to regulate host immunity. KIRs serve as recognition factors that enable NK cells to monitor tumors, infective cells, fetal cells, and damaged cells [1]. An improper functioning or expression of KIRs is associated with disease manifestations in autoimmune pathologies as well as cancer development and infectious conditions [2]. Authorities have already identified 17 KIR genes to go alongside two pseudogenes that exist on chromosome 19q13.4 [3]. KIR haplotypes exist in two primary categories, labeled A and B, depending on their contained gene compositions. The group A haplotypes contain only inhibitory KIR genes as part of their set of genes. The genes expressed in KIR haplotype A are KIR3DL3, KIR2DL3, KIR2DP1, KIR2DL1, KIR3DP1, and KIR2DL4, while the KIR haplotype B contains many diverse gene combinations between activating and inhibitory KIR genes. Various KIR haplotype B genes exist with standard components including KIR2DL5A or KIR2DL5B or both: KIR2DL1, KIR2DL2, KIR2DL3, KIR3DL1, KIR2DS4, KIR3DL2, KIR3DL3, KIR2DP1, KIR2DS2, KIR2DS3, and KIR2DS5. The KIR haplotype AB represents a combination of KIR haplotype A and B mechanisms formed by genetic recombination processes [4]. KIR haplotype AB develops diverse genes because it unites genetic components from haplotypes A and B. The A, B, and AB compositions of KIR haplotype genes show wide variation between individual subjects as well as between different populations [5]. KIR genes within chromosome 19 exist primarily in the centromeric (Cen) and telomeric (Tel) areas. Polymorphism among KIR gene region haplotypes exists at a high level due to their diverse genetic makeup. The KIR genes carry the following sets of functional and non-functional elements throughout their Cen and Tel regions. The Cen group A of haplotypes includes KIR2DL1, KIR2DL3, KIR3DL3 (inhibitory KIRs), and KIR2DS4 (activating KIR) together with the pseudogenes KIR2DP1 and KIR3DP1. Animal species possess the Tel A haplotype, which includes KIR2DL4 and KIR2DL5 with KIR2DS5 and KIR3DS1, along with the pseudogenes KIR2DP1 and KIR3DP1. The Cen group B of haplotypes includes KIR2DL2, KIR2DL5, KIR3DL2 (inhibitory KIRs), KIR2DS1 (activating KIR), and KIR2DP1 as a pseudogene. The expression group Tel B haplotype includes KIR2DL5 and KIR2DL5B alongside KIR2DS3, KIR2DS1 (activating KIRs), and the pseudogenic KIR2DP1 [6] (Table 1).

**Table 1:** The broad distribution of the contents of centromeric and telomeric A and B haplotypes of KIR genes This table was compiled and modified by the authors using information from Rajalingam [6]. KIR: killer-cell immunoglobulin-like receptor

Haplotype	Inhibitory KIR genes	Activating KIR genes	Pseudogenes
Centromeric A	KIR2DL1, KIR2DL3, KIR3DL3	KIR2DS4	KIR2DP1, KIR3DP1
Telomeric A	KIR2DL4, KIR2DL5, KIR2DS5	KIR3DS1	KIR2DP1, KIR3DP1
Centromeric B	KIR2DL2, KIR2DL5, KIR3DL2	KIR2DS1	KIR2DP1
Telomeric B	KIR2DL5, KIR2DL5B	KIR2DS3, KIR2DS1	KIR2DP1

Parham and Moffett emphasized the importance of KIR-human leukocyte antigen (HLA) interactions in shaping NK cell function, particularly within the framework of Cen and Tel KIR haplotypes. Each KIR recognizes specific HLA class I ligands, including those from the HLA class 1 groups. The Cen and Tel regions encode distinct combinations of inhibitory and activating KIR genes, with Tel regions typically enriched in activating KIRs. The presence of more activating KIRs, particularly within Tel A and Tel B haplotypes, may skew NK cell activity toward activation. However, the expression of these KIRs alone is not sufficient; their functional relevance depends on the coexpression of their specific HLA ligands. In instances where activating KIRs are present but their corresponding HLA-C ligands are absent, these receptors may not contribute to NK cell activation [7]. This receptor-ligand mismatch offers an explanation for the variability in immune responses among individuals who share similar KIR genotypes but differ in HLA backgrounds. Such diversity highlights the intelligent and adaptive nature of the immune system, in which functional outcomes are determined not just by gene content but also by receptor-ligand compatibility and NK cell subset dynamics. This variability has significant implications for understanding immune regulation and advancing personalized immunotherapies.

Several studies have evaluated the role of KIRs in disease pathogenesis [8-10]. However, most of the studies have concentrated on single KIR genes or broad haplotype classifications (e.g., A and B haplotypes), without regard to the importance of Cen and Tel KIR variations. We have shown that haplotypes were compartmentalized as a key determinant of disease susceptibility and immune outcome. When it comes to KIRs, an excessive amount of inhibitory Cen KIRs (Cen A: KIR2DL3, KIR2DL1, KIR2DP1) may contribute to immunological dysregulation in autoimmune diseases, whereas Tel activating KIRs (Tel B: KIR3DS1, KIR2DS1, KIR2DS5) are associated with enhanced immune responses during disease outbreaks [11]. Additionally, in infection, the balance between inhibitory and activating KIR haplotypes influences viral clearance or host susceptibility [12], while in reproductive immunology, maternally derived KIR receptors bind to fetal HLA-C variants, which contribute to determining the success or failure of pregnancy [13]. Additionally, the impact of donor and recipient KIR mismatches on graft acceptance, rejection, and graft-versus-host disease (GVHD) is observed in transplantation settings [14,15].

Multiple studies researched KIRs; however, researchers still need to determine the precise influence of Cen and Tel KIR variations during disease pathogenesis. All studies have focused their analysis on KIR profiles in general instead of examining the independent effects of genomic segments. The assessment of KIR haplotypes located at Cen and Tel regions needs thorough investigation since potential immune regulation effects can reveal additional insights about immune-mediated diseases. The evaluation assesses the role of KIR gene alternations in Cen and Tel regions towards several diseases categorized as autoimmune conditions, infectious agents, reproductive issues, and transplantation. Researchers demonstrate that variations in the KIR region, either towards the centromere or towards the telomere, influence susceptibility to diseases.

## Review

Linkage disequilibrium (LD) in KIR genes

LD is the non-random association of alleles at different loci, meaning certain combinations of alleles occur more (or less) frequently than expected by chance. It reflects the genetic linkage and evolutionary history of a population, including recombination, selection, and drift. LD is widely used in mapping disease genes and studying population structure [16]*. *LD between the Tel and Cen regions of KIR genes is important, particularly when studying the functions of NK cells and the variety of immunological responses. The genes within each region exhibit significant LD, indicating that they are frequently inherited as a unit. LD in genetics may be positive or negative. If KIR alleles at two non-overlapping loci coexist more than would be expected by random chance, then we find a positive LD. This suggests that they inherited the KIR alleles towards each other because they were close to each other on the chromosome. For example, negative LD is the co-occurrence of a KIR allele at one locus and its absence at another locus with a significantly reduced likelihood of co-heritability of these alleles [17]. A KIR haplotype consists of various possible combinations between Cen and Tel region variants [5]. An extensive research investigation conducted by Vierra-Green et al., shedding light on the allele-level haplotype structure and LD across the KIR gene, included 506 European-American participants. Their study, using high-resolution KIR genotyping and analysis, found a remarkable pattern of substantial intraregional LD inside the Cen and Tel areas of the KIR locus, but not between them. Particularly, their finding determined a strong LD between KIR2DL5, KIR2DS3S5, and KIR2DL1 in the Cen region. Strong LD is also present in the Tel region between KIR3DL1 and KIR2DS4 [18]. Consistently, Gourraud et al. carried out an extensive investigation to characterize the LD structure of the KIR gene cluster, utilizing a large family-based dataset comprising 418 haplotypes from 106 Northern Irish families. The analysis explored LD at two levels: structurally, in terms of gene presence or absence, and allelically, testing the relationship between specific alleles. By adopting classical LD measures such as D′, along with positive and negative predictive values (PPV and NPV), the authors identified two major LD blocks within the KIR region, that is, a Cen block (KIR3DL3-KIR2DL1) and a Tel block (KIR2DL4-KIR3DL2), isolated by a relatively low LD (D′=0.36). These blocks exhibited strong co-inherited gene coexistence patterns, and tight linkage was seen in a number of extended haplotypes. Interestingly, the study showed that six essential genes could efficiently identify the region's main structural changes, providing a simplified approach for association research. These results highlight the KIR cluster's immunogenetic complexity and offer a useful paradigm for analyzing KIR correlations in population and disease research. [19]. Understanding LD is, therefore, essential to interpreting the evolution, diversity, and functional implications of the Cen/Tel KIR framework discussed in our current review.

KIR Cen and Tel diversity

KIR genes exist on chromosome 19 in a highly changeable cluster yet exhibit different numbers of genes between regions near the chromosome centers and chromosome ends. Nakimuli et al. conducted research on the African KIR gene to determine the KIR gene diversity within Cen haplotypes [20]. It was also reported that specific KIR genes, namely, KIR2DL5, KIR2DS1, and KIR2DS2, occurred more frequently in certain population groups than in others [21].

Research conducted in Singapore shows substantial variations exist between the KIR genes of the Singapore-Chinese and Singapore-Indian populations, particularly in their Tel and Cen regions. The inhibitory KIR2DL1 gene, leading to NK cell function inhibition, occurs primarily in the Cen region of Singapore-Chinese individuals compared to Singapore-Indians. The populations have dissimilar mechanisms for controlling NK cell inhibitory functions. Singapore-Indians have activating genes KIR3DS1 and KIR2DS4 that reside primarily in the Tel region, while their Singapore-Chinese counterparts have these activating genes less frequently [22].

**Figure 1:**
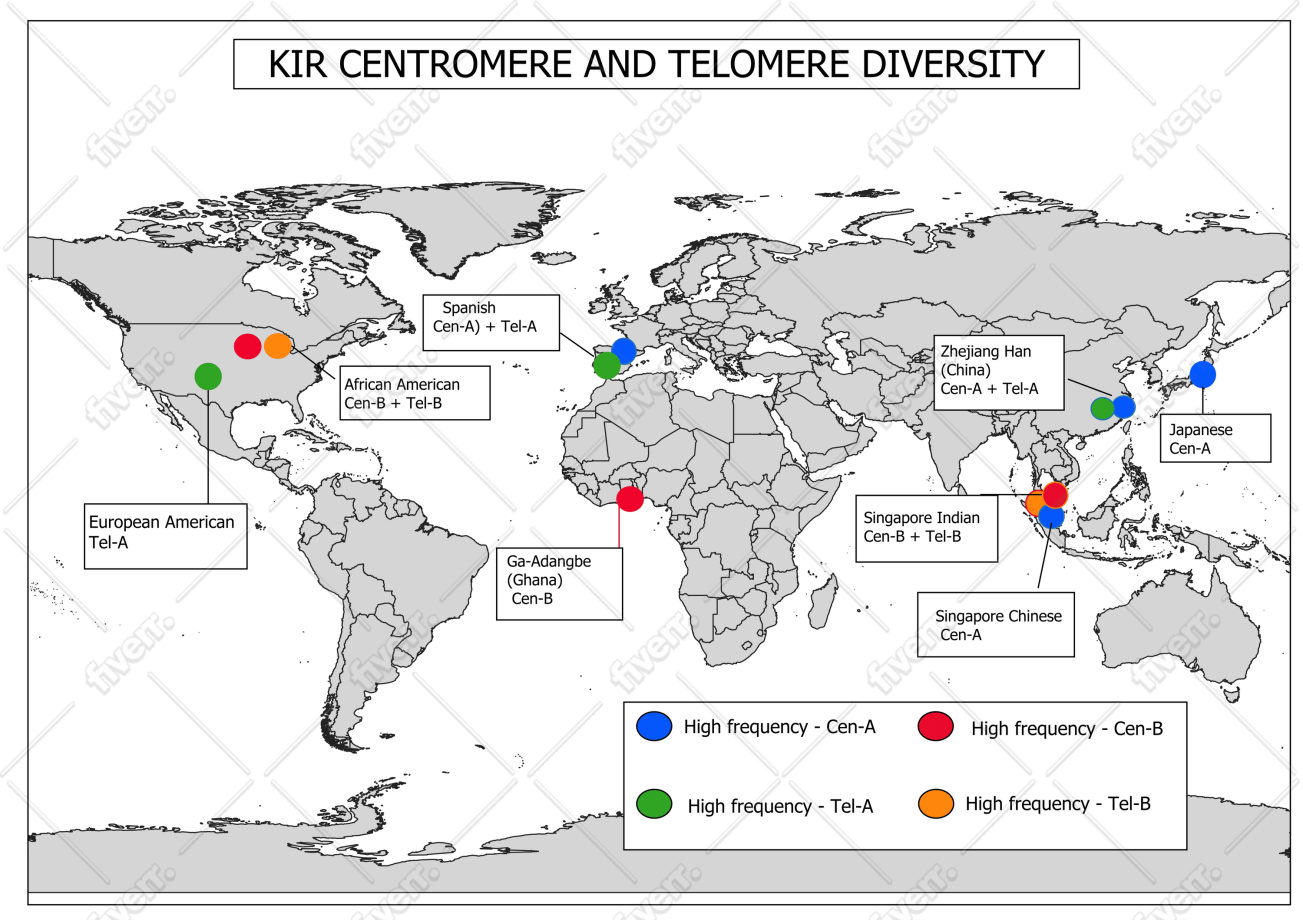
Global diversity in the distribution of Cen and Tel KIR haplotypes This world map illustrates the global diversity in the distribution of Cen and Tel KIR haplotypes, as compiled from the available literature. This figure was created by the authors using information compiled from multiple sources [18,20-23]. Cen: centromeric; Tel: telomeric; KIR: killer-cell immunoglobulin-like receptor

Amorim et al. generated a large, high-resolution study of KIR genes throughout a large North American populace. This paper focuses on how KIR genes are distributed within European-American Cen and Tel chromosome regions. Haplotype cA01, together with cB01 and cB02, forms the major haplotype composition of Cen diversity. The Tel segment of KIR genes shows two main haplotypes, including tA01 and tB01, which demonstrate one complete haplotype lacking the KIR2DS4 gene as an exception. The Tel B haplotypes (tel B) possess two distinctive alleles, KIR3DS1 and KIR2DL4, but the Tel A haplotypes (tA01) commonly have KIR3DL1 linked to other KIR gene sequences [23]. Different KIR gene contents separate African-American and European-American populations most substantially within the Tel and Cen regions. Haplotypes cA01, cB01, and cB02 represent the main variability in European-Americans' Cen region alongside tA01 and tB01 haplotypes and several new variants found at the Tel region. The higher gene duplications and deletions or recombination frequency among African-Americans exists because of their greater abundance of activating genes, specifically KIR2DS3 and KIR3DS1, in the Tel area, which produces more diversity. The copy number variant frequency of African-Americans exceeds that of European-Americans. African-Americans show various examples of KIR haplotype diversity because this creates broader immunological responses, indicating evolutionary changes [23]. The KIR gene content of Tel haplotypes in European-Americans was investigated by Vierra-Green et al., who observed dissimilarities in gene composition between haplotypes [18], as depicted in Figure 1.

Cen and Tel KIR variations in disease susceptibility

Autoimmune Diseases

The importance of KIR genes in autoimmune diseases is highlighted by the ways in which changes in their content and their interactions with HLA molecules can either protect against or enhance vulnerability to certain illnesses [8].

Rheumatoid arthritis (RA): A KIR2DS4-full gene located at the Tel section showed strong links to elevated RA risks in comparison to KIR2DL5A as the inhibitory gene of the Cen area which demonstrated protective qualities. The statistical correction process rendered the relationships between multiple haplotypes and their increased frequency association insignificant. The Cen A haplotype displayed a protective relation with RA initially but lost this statistical significance after the correction process [24].

Vogt-Koyanagi-Harada (VKH) disease: Researchers at the Ocular Inflammatory Disease Center, University of California, Los Angeles (UCLA), under Levinson and colleagues, evaluated the implications of KIR and HLA genotypes on VKH disease pathogenesis while revealing vital aspects of NK cell behavior in autoimmune condition onset. The pathogenesis of VKH disease could be caused by deficient immune surveillance because patients showed decreased expression of Cen B KIR genes, particularly 2DL2, 2DS2, 2DS3, and 2DL5B. The immune regulation of VKH patients depends on the interaction between KIR genes located at telomeres and centromeres, despite their lack of statistical differences between VKH patients and controls. The authors point out that KIR-HLA combinations, which are particularly required for NK cell education, were found to be particularly low in number, instead of the VKH patient's hyporesponsive state, allowing NK cells to be more prone to viral infection, which leads to autoimmune pathology. This study highlights the relevance of considering both Cen and Tel KIRs in attempts to comprehend the immunogenetic regulation in autoimmune disease and its possible influence as therapeutic targets [25].

Systemic lupus erythematosus (SLE): Although a lot of research has demonstrated the association between KIR gene polymorphisms and SLE [26], it is apparent that there is a gap in the literature related to KIR (Cen and Tel) haplotype analysis regarding SLE. This could be due to a limited understanding of the functional significance of synergistic effects arising from the coexpression of specific KIR receptors within a single individual. The interaction of KIRs and autoimmune diseases has been a focus for several researchers. Nevertheless, a major gap persists as these investigations tend to neglect the specificities of the Cen and Tel divisions of the KIR gene cluster.

Infectious Diseases

Most studies investigating KIRs in the context of infections have not undertaken a comprehensive analysis of the distinct roles of Cen and Tel KIR domains in regulating immune responses [27-32].

Syphilis: A research conducted by Zhuang et al. recognized the Tel B/B genotype in syphilis patients when compared to healthy controls (p=0.024). It means individuals with the Tel B/B genotype may have an increased risk of syphilis due to excessive NK cell activation and a pro-inflammatory environment, while, in contrast, the Tel A/B genotype includes both inhibitory (KIR3DL1 and KIR2DS4) and activating KIR genes, which are less frequent in syphilis patients (p=0.049). This shows a potential protective effect and a balance between inhibitory and activating KIRs in the Tel A/B genotype that provides a more regulated immune response, which can control syphilis susceptibility. Research results showed no differences in syphilis patients versus healthy subjects regarding their Cen KIR genotypes (Cen A/A, Cen A/B, Cen B/B), indicating this region played an insignificant role in susceptibility to the disease for this population [33].

Post-hematopoietic stem cell transplantation (post-HSCT)-associated infection: Bultitude et al. found that donor-encoded Cen B motifs were significantly associated with a greater risk of non-relapse mortality (NRM) one year following hematopoietic cell transplantation (HCT) for acute myeloid leukemia (AML) (p=0.001). Infections represent the most common cause of death among these patients, especially highlighting viral diseases. The expression of Cen B motif donors leads to increased infection-related deaths during one-year follow-up after transplantation as compared to Cen A donors (Cen AA: 6% vs. Cen Bx: 25%; p=0.006). Higher Cen B motif expression showed a dose-dependent correlation with NRM risk. The research suggests that KIR2DL2/3 alleles with increased binding to HLA-C ligands create stronger NK cell blockage that ends up reducing viral clearance within T-cell-depleted (TCD) transplants where T-cell immunity is compromised. The KIR2DL2/3 alleles, which have lower affinity for Cen A binding, create an advantage against viral infection by enabling NK cell activation more readily. Tel B KIR motifs failed to show any meaningful correlation with NRM or infection-related mortality according to this study, indicating that the Cen segment plays an essential role in regulating NK cell responses against viral infection in this situation [34].

These findings highlight that while Tel KIR genotypes may influence bacterial infection susceptibility (e.g., syphilis), Cen motifs might play a more prominent role in viral immunity, especially under conditions of impaired adaptive responses. This underscores the need for future infection-related studies to dissect Cen-/Tel-specific contributions to NK cell regulation more systematically.

Transplantation Outcomes 

The essential role of NK cells in immune regulation and graft recognition, which depends on KIR signaling, had been previously documented [35-37]. Particularly, the contribution of Cen and Tel KIR haplotypes in allogeneic HSCT has become increasingly recognized for its influence on post-transplant outcomes. A growing body of evidence supports the clinical utility of KIR haplotype analysis, particularly in donor selection strategies aimed at optimizing survival, reducing relapse, and minimizing GVHD. A previous study by Ruggeri et al. provided compelling clinical evidence for the beneficial role of donor NK cell alloreactivity in HLA-mismatched HSCT [38]. The study demonstrated that NK cell-mediated graft-versus-leukemia (GvL) effects arising from KIR-HLA mismatches in the graft-versus-host direction significantly reduced relapse rates in patients with AML. Moreover, transplantation from NK alloreactive donors was associated with enhanced engraftment and a lower incidence of GVHD. These findings highlighted the clinical relevance of KIR-HLA interactions and contributed to the growing interest in incorporating NK cell alloreactivity, through donor KIR typing and recipient HLA ligand analysis, into donor selection strategies for mismatched HSCT, particularly in AML cases.

Zhou et al. examined outcomes in HLA-identical sibling HSCT and reported that the presence of donor Cen B motifs was significantly associated with improved overall survival (OS) and relapse-free survival (RFS), whereas Tel B haplotypes showed no significant clinical impact. Their study also demonstrated that specific donor-recipient KIR mismatches in AML enhanced GVL effects while mitigating severe acute GVHD, reinforcing the value of integrating Cen B haplotype screening into donor selection protocols [39].

Cooley et al. provided additional support by demonstrating that donors with Cen B/B genotypes were associated with lower relapse incidence and better OS and RFS in AML patients undergoing unrelated HSCT. Importantly, these benefits were achieved without increased GVHD risk, emphasizing the relevance of B-content KIR genotyping in donor selection [40]. Consistently, a study by Bao et al. highlighted that donor KIR B haplotypes with Cen B-specific motifs (Cen B) significantly improved OS and RFS and reduced NRM in standard-risk AML/myelodysplastic syndrome (MDS) patients undergoing unrelated donor HSCT. This positive effect was independent of Cen B motif subtypes. In contrast, the classical B haplotype profile was associated with poorer outcomes. These findings support incorporating Cen B presence into donor selection and avoiding classical B haplotyping profiles for standard-risk cases [41].

Dubreuil et al. highlighted the clinical relevance of the Cen AA (cenAA) KIR haplotype in haploidentical HSCT. Donors with cenAA carried favorable inhibitory alleles (e.g., KIR2DL1003, KIR2DL3001) associated with enhanced NK cell education and function. This was linked to reduced relapse risk in myeloid malignancies without increasing GVHD, emphasizing cenAA's immunoregulatory advantage and its value in donor selection [38,42].

In pediatric transplantation, Rangarajan et al. studied 165 juvenile myelomonocytic leukemia (JMML) patients and found that donors with Cen A/B or Tel A/B configurations were associated with a significantly lower risk of grade II-IV acute GVHD (HR=0.57 and HR=0.58, respectively). While OS and relapse rates did not differ significantly by haplotype group, the protective effect against GVHD underscores the immunomodulatory contribution of Cen/Tel combinations [43].

A comprehensive research review about the role of KIR in allogeneic HSCT by Dhuyser et al. analyzed various models predicting NK cell alloreactivity in HSCT and emphasized the lack of a unified donor selection strategy. The study highlighted that Cen B motifs showed the strongest association with reduced relapse and better survival. Outcomes varied across transplant protocols and populations. The benefit of NK alloreactivity was most evident in TCD settings. The authors recommend integrated scoring models for improved donor selection [44].

Collectively, these findings affirm that the Cen/Tel KIR haplotype framework is not only a valuable predictor of transplant success but also an essential tool for refining donor selection criteria across diverse transplantation settings.

Reproductive Health

Researches confirmed that NK cells function as the primary immune cells present in the uterus [45]. Numerous scientists have studied KIRs because these receptors act as essential receptors on NK cells during pregnancy and associated complications. Various studies in the past linked KIR with HLA in pregnancy complications, yet most analyses omitted the investigation of KIR Cen and Tel haplotypes because researchers studied only classical KIR haplotypes. According to our unpublished findings about HLA and KIR involvement in recurrent miscarriages, which included 68 patients with recurrent miscarriages and 91 healthy control group, we observed that patients with recurrent miscarriages displayed increased Cen BB frequency, which exceeded the healthy control group frequency at p=0.001 with an odds ratio of 8.31. We also reported that the Cen AB KIR haplotype presented at elevated levels within the control group, thus suggesting that surplus NK cell activation could result in pregnancy loss. Furthermore, we did observe that women who expressed homozygous Cen BB haplotypes had higher chances of pregnancy loss in comparison to the control group, particularly those carrying homozygous HLA-C2C2. However, the coexistence of the Cen AB haplotype with heterozygous HLA-C1C2 was common in healthy control women, so it is suggested to be associated with protection against recurrent miscarriage. The research by Hiby and colleagues documented how women with the KIR AA haplotype genotype suffered recurrent miscarriage compared to subjects with BB or AB genotype in a different population group. They also demonstrate that women with lower expression of Tel B haplotypes showed a reduced risk of pregnancy loss, although the association did not reach statistical significance. Notably, the frequency distribution of Tel B was lower among healthy control women compared to those with adverse pregnancy outcomes [46]. A recent case-control study on the Spanish population involving 397 women and their partners demonstrated extensive investigation for both Cen and Tel KIR haplotypes as essential factors for pregnancy success and complications. They documented that the Cen AA KIR haplotype serves as a significant biomarker for alloimmune reproductive failure (uRF) that includes recurrent pregnancy loss (RPL) and recurrent implantation failure (RIF). Gil Laborda et al. propose that the classification of KIR haplotypes based on Cen and Tel regions provides better predictive value than traditional KIR classifications, particularly in identifying women at increased risk within the heterogeneous KIR AB or Bx populations [47]. These research outcomes support the hypothesis that KIR interactions between maternal and fetal cells promote successful pregnancies while showing the necessity for customized immunology treatments in reproduction (Table 2). We propose that KIR haplotype screening, particularly the characterization of Cen and Tel motifs, could be integrated into reproductive immunology practice as a complementary tool in risk stratification for pregnancy complications such as recurrent miscarriage or implantation failure. Screening could be particularly valuable in couples undergoing assisted reproductive technologies (ART), where matching maternal KIR haplotypes with paternal or fetal HLA-C genotypes may help identify at-risk pregnancies. This approach could inform personalized immunological monitoring or the development of targeted interventions. However, standardized guidelines and further validation in large, diverse cohorts are essential before clinical implementation.

**Table 2:** Cen and Tel KIR variations in disease susceptibility This table was created by the authors with data compiled from multiple sources. Relevant citations are provided within the table content. Cen: centromeric; Tel: telomeric; KIR: killer-cell immunoglobulin-like receptor; HSCT: hematopoietic stem cell transplantation; RA: rheumatoid arthritis; VKH: Vogt-Koyanagi-Harada; NK: natural killer; SLE: systemic lupus erythematosus; NRM: non-relapse mortality; OS: overall survival; RFS: relapse-free survival; GVL: graft-versus-leukemia; aGVHD: acute graft-versus-host disease; RM: recurrent miscarriage; uRF: alloimmune reproductive failure; RPL: recurrent pregnancy loss; RIF: recurrent implantation failure

Disease category	Cen KIR association	Tel KIR association
Autoimmune diseases	The Cen A haplotype was initially linked to protection in RA but lost significance after correction [24].	The Tel B activating gene KIR2DS4-full increases RA risk [24].
	Cen-B reduction in VKH disease was linked to lower NK cell response [25].	There is no significant difference in Tel KIRs in VKH disease, but interaction with Cen KIRs is crucial [25].
	Studies on KIR-SLE association exist, but specific Cen vs. Tel effects remain unexplored [8].	There is a lack of research on Tel KIRs in SLE, but the coexistence of some KIRs may influence NK cell activity [26].
Infections	Cen B motifs in donors increase NRM post-HCT, mainly due to viral infections. Higher Cen B copies correlate with increased NRM risk [34].	The Tel B/B genotype (KIR3DS1, KIR2DS1, KIR2DS5) increases syphilis risk. Tel A/B (with inhibitory KIRs) is protective. There is no association between Cen KIRs and syphilis [33].
HSCT	Cen B and Cen B/B are associated with improved OS and RFS and reduced relapse, predictive of better outcomes across HSCT settings [39-41,44].	Tel B haplotypes (KIR2DS3, KIR2DS1, KIR3DS1) are linked to better GVL effects and reduced relapse [44].
	Cen AA (with inhibitory alleles) is linked to reduced relapse risk in myeloid malignancies [42].	
		Tel A/B is also associated with reduced GVHD risk [43].
	Cen A/B is associated with reduced risk of grade II-IV aGVHD [43].	
Pregnancy and reproductive immunology	Cen BB haplotype is significantly higher in RM patients (unpublished data).	The frequency distribution of Tel B was lower among healthy control women compared to those with adverse pregnancy outcomes [46].
	Cen AA increases pregnancy complications (uRF, RPL, RIF) [47].	

## Conclusions

This review centered around those studies that examine the correlation between the KIR Cen and Tel haplotypes with respect to disease susceptibility. We purposely omitted studies focusing on individual KIR alleles and classical haplotypes because these have been sufficiently covered in the literature. We wish to emphasize the potential importance of the concomitant expression of particular KIR genes because of LD, which is recognized as Cen and Tel KIR haplotypes. Understanding the Cen-Tel framework of KIR haplotypes could expand knowledge about disease immunity regulation, disease predisposition, and clinical treatment for autoimmune disorders, infections, transplantation situations, and pregnancy conditions. Evidence suggests that Cen B haplotypes are associated with increased risk in conditions such as RA, HCT-related mortality, and pregnancy loss, while Tel B haplotypes exhibit both protective and pathogenic roles depending on the disease context. Notably, Cen B motifs have been linked to enhanced GVL effects and reduced relapse in HSCT, whereas Tel B profiles may support graft tolerance in solid organ transplantation. Similarly, in reproductive immunology, Cen-Tel diversity influences maternal-fetal tolerance and risk of RPL. Despite these findings, many studies fail to distinguish between the Cen and Tel contributions of KIR polymorphisms, representing a critical gap in current immunogenetic research. Future investigations that integrate Cen and Tel KIR haplotype analysis could refine risk stratification, optimize donor selection in transplantation, and advance personalized therapies in infectious, autoimmune, and reproductive disorders.
